# Morris Water Maze Training in Mice Elevates Hippocampal Levels of Transcription Factors Nuclear Factor (Erythroid-derived 2)-like 2 and Nuclear Factor Kappa B p65

**DOI:** 10.3389/fnmol.2015.00070

**Published:** 2015-11-18

**Authors:** Wanda M. Snow, Payam S. Pahlavan, Jelena Djordjevic, Danielle McAllister, Eric E. Platt, Shoug Alashmali, Michael J. Bernstein, Miyoung Suh, Benedict C. Albensi

**Affiliations:** ^1^Division of Neurodegenerative Disorders, St. Boniface Hospital ResearchWinnipeg, MB, Canada; ^2^Faculty of Health Sciences, Department of Pharmacology and Therapeutics, College of Medicine, University of ManitobaWinnipeg, MB, Canada; ^3^Department of Human Nutritional Sciences, University of ManitobaWinnipeg, MB, Canada; ^4^Department of Psychological and Social Sciences, Pennsylvania State University AbingtonAbington, PA, USA; ^5^Faculty of Engineering, Department of Electrical and Computer Engineering, University of ManitobaWinnipeg, MB, Canada

**Keywords:** spatial memory, activity-dependent plasticity, transcription factors, Morris water maze, cAMP-response element binding protein, nuclear factor (erythroid-derived 2)-like 2, nuclear factor kappa B, early growth response-2

## Abstract

Research has identified several transcription factors that regulate activity-dependent plasticity and memory, with cAMP-response element binding protein (CREB) being the most well-studied. In neurons, CREB activation is influenced by the transcription factor nuclear factor kappa B (NF-κB), considered central to immunity but more recently implicated in memory. The transcription factor early growth response-2 (Egr-2), an NF-κB gene target, is also associated with learning and memory. Nuclear factor (erythroid-derived 2)-like 2 (Nrf2), an antioxidant transcription factor linked to NF-κB in pathological conditions, has not been studied in normal memory. Given that numerous transcription factors implicated in activity-dependent plasticity demonstrate connections to NF-κB, this study simultaneously evaluated protein levels of NF-κB, CREB, Egr-2, Nrf2, and actin in hippocampi from young (1 month-old) weanling CD1 mice after training in the Morris water maze, a hippocampal-dependent spatial memory task. After a 6-day acquisition period, time to locate the hidden platform decreased in the Morris water maze. Mice spent more time in the target vs. non-target quadrants of the maze, suggestive of recall of the platform location. Western blot data revealed a decrease in NF-κB p50 protein after training relative to controls, whereas NF-κB p65, Nrf2 and actin increased. Nrf2 levels were correlated with platform crosses in nearly all tested animals. These data demonstrate that training in a spatial memory task results in alterations in and associations with particular transcription factors in the hippocampus, including upregulation of NF-κB p65 and Nrf2. Training-induced increases in actin protein levels caution against its use as a loading control in immunoblot studies examining activity-dependent plasticity, learning, and memory.

## Introduction

The formation of various forms of memory is regulated by distinct neurobiological mechanisms. For example, the formation of long-term (across hours to days), but not short-term (across minutes), memory is associated with protein synthesis (Davis and Squire, 1984; Kandel, 2001; Alberini, 2009). Transcription factors, which can either repress or activate transcription, play a vital role in driving protein synthesis underlying synaptic plasticity and memory, whereby protein synthesis provides the necessary building blocks to accommodate structural changes at the synapse that foster memory formation (Alberini, 2009; Alberini and Kandel, 2014).

The transcription factor cAMP-response element binding protein (CREB) was among the first to be examined in a context of memory, starting with seminal work linking CREB-mediated gene expression to long-term facilitation of the gill-withdrawal reflex in the invertebrate *Aplysia* (Dash et al., 1990; Kaang et al., 1993; Bartsch et al., 1995). The importance of CREB in long-term memory (LTM) has also been demonstrated in *Drosophila melanogaster* (Yin et al., 1994) as well as mammals, including mice (Bourtchuladze et al., 1994) and rats (Josselyn et al., 2001). These data, therefore, suggest a phylogenetically conserved role for CREB in LTM formation.

The consolidation of long-term spatial memories requires protein synthesis and is generally considered to be CREB-dependent (Benito and Barco, 2010). For example, CREB knockout (KO) mice exhibit deficits in learning the location of a hidden platform based on visual cues (Bourtchuladze et al., 1994) in the Morris water maze (MWM), a behavioral paradigm to assess rodent spatial learning and memory (Morris et al., 1982). These mice also display deficits in recalling the platform location after 15 days of training. In rodents, spatial memory formation and intact MWM performance rely critically on the hippocampus (Morris et al., 1982; Bannerman et al., 1999). In rats, disruption of hippocampal CREB via antisense oligodeoxynucleotides impairs long-term spatial memory formation in the MWM (Guzowski and McGaugh, 1997). Further, hippocampal CREB levels have been shown to be strongly correlated with spatial memory capabilities in mice (Brightwell et al., 2004).

In addition to CREB, other transcription factors implicated in memory have been identified (Alberini, 2009; Alberini and Kandel, 2014), including nuclear factor kappa B (NF-κB)(Snow et al., 2014). NF-κB belongs to the Rel family, consisting of five members that form various dimers: p50, p52, p65/RelA, RelB, and c-Rel (Alberini, 2009). Only p65, c-Rel, and RelB, however, have transcriptional activation domains in the C-terminal region to induce transcription, whereas homodimers consisting of p50 and p52 suppress gene expression (Ghosh and Karin, 2002). In neurons, the most common dimers include the p50 homodimer and p65-p50 heterodimer (Meberg et al., 1996). These dimers reside in the cytoplasm in an inactive state, where they are bound to inhibitory IκB proteins. Upon stimulation, phosphorylation of the IκB subunit by IκB kinase (IKK) targets it for degradation by the proteasome, freeing the dimer to translocate to the nucleus where it regulates the expression of genes with DNA-binding sites for NF-κB (Alberini, 2009). Several activators of neuronal NF-κB have been identified, including tumor necrosis factor (Albensi and Mattson, 2000), glutamate, nerve growth factor (Meffert and Baltimore, 2005), dopamine, nitric oxide, kainite (Simpson and Morris, 1999), calcium (Cruise et al., 2000), NMDA receptor activation (Burr and Morris, 2002), and excitatory synaptic transmission via a Ca^2+^-dependent process (Alberini, 2009). Further, the induction of long-term potentiation (LTP), a cellular correlate of learning and memory, is associated with NF-κB activation, resulting in an increase in the p65-p50 heterodimer and a decrease in IκB mRNA (Meberg et al., 1996). In the crab *Chasmagnathus, in vivo* experiments demonstrate increased activation of NF-κB in brain cell nuclei after a fear stimulus (Freudenthal et al., 1998). Further, injection of IKK blocks its activation, disrupting memory formation (Merlo et al., 2005). Inhibition of NF-κB reduces neural growth and branching in the hippocampus (O'sullivan et al., 2010). Moreover, p50-KO mice demonstrate deficits in late-phase LTP as well as selective deficits in spatial memory assessed in the MWM (Oikawa et al., 2012). Downregulation of neuronal NF-κB results in decreased activation of CREB via alterations in protein kinase A (Kaltschmidt et al., 2006), which activates CREB by phosphorylation at Serine 133 (Walton and Dragunow, 2000).

More recently, the early growth response (Egr) family of transcription factors, consisting of Egr1-4, has been implicated in memory. The roles of these various members in memory, however, are not well-defined. For example, Egr-1 is specific to spatial navigation long-term memory consolidation (Jones et al., 2001), whereas Egr-3 is implicated in short-term acquisition (Li et al., 2007). Other studies, however, have reported improved memory in Egr-2 KO animals (Poirier et al., 2007). Egr-2 has inhibitory roles on certain cognitive functions via regulating the expression of Nab1 and Nab2 proteins (Desmazières et al., 2008) that increase transcriptional activity of other Egr family where their effect is through Nab, resulting in facilitation in some types of memory (Poirier et al., 2008). In neurons, Egr-2 is a downstream target of NF-κB (Nafez et al., 2015). As with NF-κB, studies suggest a role for Egr-2 in establishing persistent LTP (Williams et al., 1995), consistent with a role for this isoform in learning and memory.

The transcription factor nuclear factor (erythroid-derived 2)-like 2 (Nrf2) is a key regulator of antioxidant genes. Encoded by the *NFE2L2* gene, Nrf2 resides in the cytoplasm under basal conditions. Cellular stressors (i.e., oxidative, electrophilic), however, activate Nrf2, resulting in its translocation to the nucleus where it forms a heterodimer with Maf protein and initiates gene transcription through binding to DNA promoter regions (Tebay et al., 2015). Several studies report crosstalk between Nrf2 and NF-κB in pathological conditions, including cellular exposure to methamphetamine (Permpoonputtana and Govitrapong, 2013) as well as experimental models of diabetes (Agca et al., 2014), Alzheimer's disease (Ashabi et al., 2013), chronic stress (Djordjevic et al., 2015), spinal cord injury (Jin et al., 2014), and Parkinson's disease (Tobón-Velasco et al., 2013). Environmental enrichment elevates Nrf2 levels in the hippocampi of rats that have undergone experimental induction of cerebral hypoperfusion, a model of vascular dementia (Yang et al., 2015). These results suggest a role for Nrf2 in cognitive recovery after brain damage. Despite the identified role of NF-κB in synaptic plasticity, learning, and memory and its relationship to Nrf2 under several pathological conditions, alterations in Nrf2 in the context of activity-dependent plasticity, learning and memory in the typical, intact mammalian brain have not been investigated.

To further elucidate the transcriptional regulators of activity-dependent plasticity, this research investigated the effects of training in a spatial learning and memory task on transcriptional regulation in the hippocampus. We hypothesized that MWM training would alter levels of and/or be associated with NF-κB as well as transcription factors previously shown to interact (directly or indirectly) with NF-κB, specifically Nrf2, CREB (total and phosphorylated), and Egr-2, in the CD1 mouse hippocampus. Given recent technical reports questioning the use of actin as a loading control for normalization of Western blot data due to studies documenting experimentally-induced changes in actin levels (Gilda and Gomes, 2013; Li and Shen, 2013; Rivero-Gutierrez et al., 2014), we investigated putative differences in actin as a function of MWM-training in the mouse hippocampus. Here, we report several novel findings, including upregulation of Nrf2 and actin in the hippocampi of MWM-trained mice relative to untrained controls. Further, Nrf2 was highly correlated with performance in the memory retention assessment of the MWM in nearly all tested animals, suggesting a newly-identified role for Nrf2 in activity-dependent plasticity, learning, and memory outside of antioxidant regulation in the intact mammalian brain. This is the first report, to our knowledge, detailing parallel upregulation of both p65 NF-κB subunit and Nrf2 after training, in contrast to prior studies documenting opposing regulation under pathological and/or inflammatory conditions.

## Materials and methods

### Animals

Experiments were carried out in 1 month-old male CD1 mice (*N* = 20) purchased from Jackson Laboratory (Bar Harbor, ME, USA). Mice were housed in the pathogen-free animal facility at St. Boniface Research Centre and maintained on a 12-h light/12-h dark cycle at room temperature (22°C). Food and water were provided *ad libitum*. All procedures were approved by the University of Manitoba Animal Care and Use Committee, which adheres to the guidelines set forth by the Canadian Council on Animal Care. One half of the mice (*n* = 10) underwent MWM training.

### MWM training

The MWM was used to assess hippocampal-dependent spatial memory using methods previously described (Kaltschmidt et al., 2006; Kishida et al., 2006; Oikawa et al., 2012). The standard MWM consisted of a circular pool (100 cm diameter) filled with water (24–25°C) made opaque (white) with powdered milk. Visual cues were positioned equidistant above the water level, and unwanted extra-maze cues were blocked with a curtain. A non-visible escape platform (7 cm diameter) was submerged ~5 mm below the water surface in the center of the designated target quadrant. In the acquisition phase (4 trials/day for 6 consecutive days), mice were given up to 60 s per trial to find the hidden platform and were required to remain seated on the platform for 10 s, after which the mice were returned to their home cage. Live video was recorded for each trial using the Videomex tracking system (Columbus Instruments, Columbus, OH, USA). Escape latency data (i.e., time to locate the platform) were extracted from video data during the acquisition phase. Search strategies were assigned for every trial of the acquisition phase using the classification scheme, as per Brody and Holtzman (2006). The search strategy employed by an animal in the MWM varies across training days and can indicate the formation of a spatial map (Guzowski and McGaugh, 1997; Kishida et al., 2006; Poirier et al., 2007). For example, during early training, mice will exhibit wall-hugging but tend to display a more focused search of the platform across training, representing the use of a spatial search strategy. Therefore, search strategies employed during the MWM were categorized into three main strategies, as previously described (Guzowski and McGaugh, 1997; Poirier et al., 2007): (1) repetitive looping: swimming in a circular pattern approximately equidistant from the pool wall (chaining), swimming in a circular pattern along the periphery of the pool (peripheral looping), swimming in tight circular patterns (circling), and/or thigmotaxis (wall hugging); (2) non-spatial systemic: searching the interior portion of the pool without an apparent spatial focus (scanning), searching the entire pool randomly without an apparent spatial focus (random), and/or searching a defined area of the pool in an incorrect quadrant; and (3): spatial strategies: swimming directly to the platform (spatial direct), swimming to the platform without repeated looping (spatial indirect), or swimming directly to the correct target quadrant, with continued searching of the platform confined to the target quadrant. The strategy that best described the majority of the swim path was assigned to each trial. During the retention phase, the platform was removed from the pool, and each mouse was given up to 60 s to search for the position of the missing platform (4 trials/day for 3 days). Several parameters were extracted from retention phase data, including time spent in the target quadrant, time spent in non-target quadrants, and the number of passes over the missing platform location.

### Brain tissue collection

The day following training, mice were sacrificed by isoflurane inhalation, followed by decapitation. Brains were rapidly excised and hippocampi extracted. Hippocampi were placed in Hibernate (Gibco) and weighed. Tissues were snap-frozen in liquid nitrogen and stored at −80°C prior to Western blotting.

### Protein extraction

Hippocampal tissue was homogenized in ice-cold RIPA buffer (150 mM sodium chloride, 1.0% Triton X-100, 0.5% sodium deoxycholate, 0.1% sodium dodecyl sulfate (SDS), and 50 mM Tris, pH 8.0) supplemented with 1% protease inhibitor cocktail (Amresco, Solon, OH, USA) and 1% phosphatase inhibitor cocktail (Sigma-Aldrich, St. Louis, MO, USA). To further solubilize intracellular proteins, samples were incubated with constant agitation for 35–40 min at 4°C. Tissue lysates were then centrifuged at 10,000 rpm for 10 min (4°C) and supernatants collected. Protein concentrations were estimated using the DC Protein Assay (Bio-Rad, Hercules, CA, USA) as described by the manufacturer. Subsequently, samples were diluted to an equal concentration with RIPA buffer.

### Western blotting

To prepare the tissue lysates for Western blotting, a 4X Laemmli buffer (16% SDS, 40% glycerol, 20% β-mercaptoethanol, 0.01% bromophenol blue, and 0.25 M Tris, pH 6.8) was added. Prior to electrophoresis, samples were denatured at 95°C for 8 min. Fifteen micrograms of protein from each sample was separated by SDS-PAGE at 200 V for approximately 45 min with 10% Criterion™ Tris-Glycine eXtended (TGX) Stain-Free™ polyacrylamide gels (Bio-Rad, Hercules, CA, USA). Stain-Free™ gels were activated by UV transillumination for 2.5 min using the ChemiDoc™ MP (Bio-Rad, Hercules, CA, USA). Proteins were transferred to nitrocellulose membranes (Bio-Rad, Hercules, CA, USA) by the Trans-Blot® Turbo™ Transfer System (Bio-Rad, Hercules, CA, USA). Transfer efficiency was visualized with the ChemiDoc™ MP. The nitrocellulose membranes were then blocked for 1 h at room temperature with 5% skim milk in 1X Tris-buffered saline with 0.1% Tween-20 (TBS-T), except those for which pCREB was detected, in which case 5% bovine serum albumin (BSA) in 1X TBS-T was used to reduce non-specific binding. After blocking, membranes were incubated overnight at 4°C with the following diluted primary antibodies: rabbit monoclonal anti-pCREB (detects CREB phosphorylated at Serine 133) (molecular weight (MW): 37 kDa; 1:1000 dilution, Abcam, Cambridge, UK, cat. no. ab32096), rabbit monoclonal anti-NF-κB p50/p105 (MW: 50 kDa/105 kDa; 1:5000 dilution, Abcam, Cambridge, UK, cat. no. ab32360), rabbit polyclonal anti-NF-κB p65 (MW: 60 kDa; 1:2000 dilution, Abcam, Cambridge, UK, cat. no. ab16502), rabbit polyclonal anti-Nrf2 (MW: 61kDa; 1:100 dilution, Santa Cruz Biotechnology, Dallas, TX, USA, sc-13032), and rabbit monoclonal anti-Egr-2 (a.k.a. krox-20, cat. no. ab108399) (MW: 53 kDa; 1:7500 dilution, Abcam, Cambridge, UK). Following incubation with primary antibodies, nitrocellulose membranes were washed with 1X TBS-T and then incubated with a peroxidase-conjugated AffiniPure goat anti-rabbit IgG (H + L) antibody (1:2000 dilution, Jackson ImmunoResearch Laboratories, West Grove, PA, USA) for 1 h at room temperature. After washing with 1X TBS-T, the relative amount of bound antibody was measured using enhanced chemiluminescence (ECL). Proteins of interest were detected using the Bio-Rad Clarity™ Western ECL Blotting Substrate (Bio-Rad, Hercules, CA, USA) and visualized by the ChemiDoc™ MP (Bio-Rad, Hercules, CA, USA) with ImageLab™ software. Membranes probed for pCREB were stripped and re-probed with the rabbit monoclonal anti-CREB (MW: 37 kDa; 1:1000 dilution, Abcam, Cambridge, UK, cat. no. ab32515) primary antibody. A subset of membranes was also stripped and re-probed with the rabbit polyclonal anti-actin primary antibody (MW: 42 kDa; 1:500 dilution, Sigma-Aldrich, St. Louis, MO, USA, cat. no. A5060). Band intensities were quantified using ImageLab™ software and normalized to the total amount of protein per lane.

### Statistical analyses

Behavioral data from the acquisition phase of the MWM were analyzed by One-way repeated-measures analysis of variance (ANOVA) across Days (6), followed by *post-hoc* comparisons using Fisher's least significant difference (LSD) tests. Analyses were conducted on the mean scores/day from the four daily trials. Search strategy data were analyzed using Chi-square tests of the proportion of trials for which a given strategy was employed. Data were analyzed using paired samples *t*-tests to compare the time spent in the target quadrant relative to the average time spent in the three non-target quadrants during the retention phase. Western blot data were analyzed using student's *t*-tests, as all variables followed a normal distribution. To investigate the relationship between transcription factor levels and memory formation in the MWM, Pearson correlations were performed on band densitometry data and retention phase parameters, including cumulative number of passes over the missing platform area and time in target quadrant. Significance was predetermined at *p* < 0.05, and all analyses were two-tailed.

## Results

### MWM-acquisition phase

**Figure 1:**
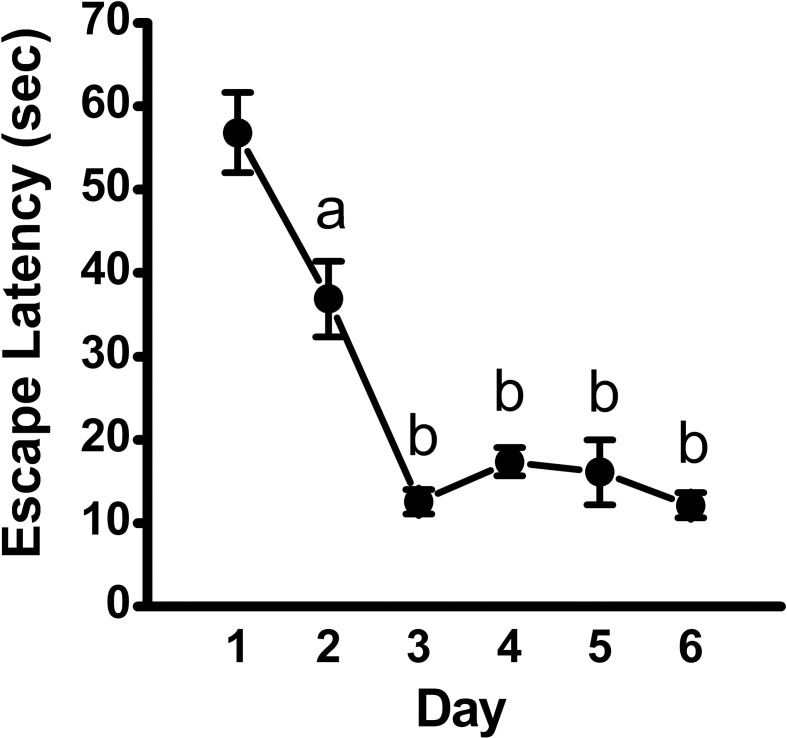
**Escape latency across training days in the acquisition phase of the MWM**. Plot depicting time to locate the hidden platform (mean ± SEM; *n* = 10) across training days, as assessed using repeated-measures ANOVA, followed by Fisher's LSD *post-hoc* comparisons. a, significantly different from Day 1, *p* < 0.01; b, significantly different from Day 1 and Day 2, *p* < 0.001.

**Figure 2:**
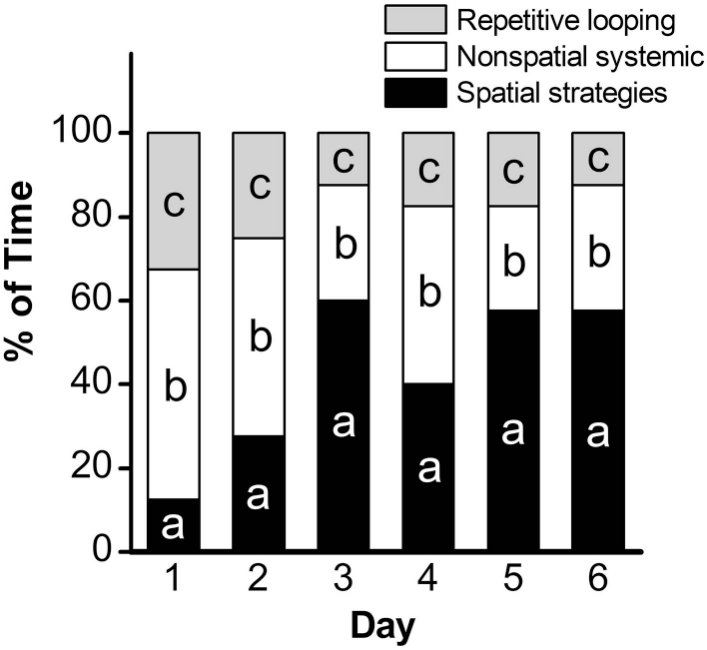
**Assessment of search strategy in the acquisition phase of the MWM**. The percentage of time engaged in specific search strategies during the 60-s trial was calculated, with search strategies combined into 3 groups based on functional similarity (repetitive looping, nonspatial systemic strategies, and spatial strategies) and analyzed using Chi Square. a: *p* < 0.001; b: *p* = 0.001; c: *p* = 0.02. *n* = 10.

Analysis of escape latency, the time the mouse took to find the hidden platform, revealed a significant decrease over time in the acquisition phase [repeated measures ANOVA; *p* < 0.001, *F*_(5, 45)_ = 34.95]. *Post-hoc* comparisons revealed a significant decrease in escape latency as early as Day 2 (*p* < 0.01), with continued improvements at Day 3 (vs. Day 2: *p* < 0.001; vs. Day 1: *p* < 0.001; Figure 1). Overall, mean latency (±SEM) decreased from 56.9 ± 4.8 s on Day 1 to a mean of 12.1 ± 1.5 s on Day 6 (*p* < 0.001), suggestive of learning the platform location. Search strategy data, analyzed using a 3 (Spatial Strategy) × 6 (Day) Chi-square analysis, revealed overall differences in the frequencies across Day and Strategy, $\chi_{\left(10\right)}^{2}=78.78,p<0.001$. Additional analyses revealed that search strategy differed at Day 1, $\chi_{\left(2\right)}^{2}$ = 27.13, *p* < 0.001; Day 2, $\chi_{\left(2\right)}^{2}$ = 9.13, *p* = 0.01; Day 3, $\chi_{\left(2\right)}^{2}$ = 35.38, *p* < 0.001; Day 4, $\chi_{\left(2\right)}^{2}$ = 11.38, *p* = 0.003; Day 5, $\chi_{\left(2\right)}^{2}$ = 27.13, *p* < 0.001; and Day 6, $\chi_{\left(2\right)}^{2}$ = 30.88, *p* < 0.001. For spatial strategies, a significant difference in the frequency of use occurred across days, $\chi_{\left(5\right)}^{2}$ = 44.41, *p* < 0.001; differences also emerged for non-spatial strategies, $\chi_{\left(5\right)}^{2}$ = 19.59, *p* = 0.001, and repetitive looping, $\chi_{\left(5\right)}^{2}$ = 15.59, *p* = 0.02. Mice used a mixture of strategies on the first day, including spatial (12.5%), non-spatial (55%), and repetitive looping (32.5%) and then showed a progressive increase (day 2, 27.5%; day 3, 60%; day 4, 40%; day 5, 57.5%; day 6, 57.5%; in the use of spatial strategies over the rest of the 6-day acquisition phase (Figure 2). This increased reliance on spatial strategies to locate the platform is consistent with the formation a cognitive spatial map (Brody and Holtzman, 2006).

### MWM-retention phase

**Figure 3:**
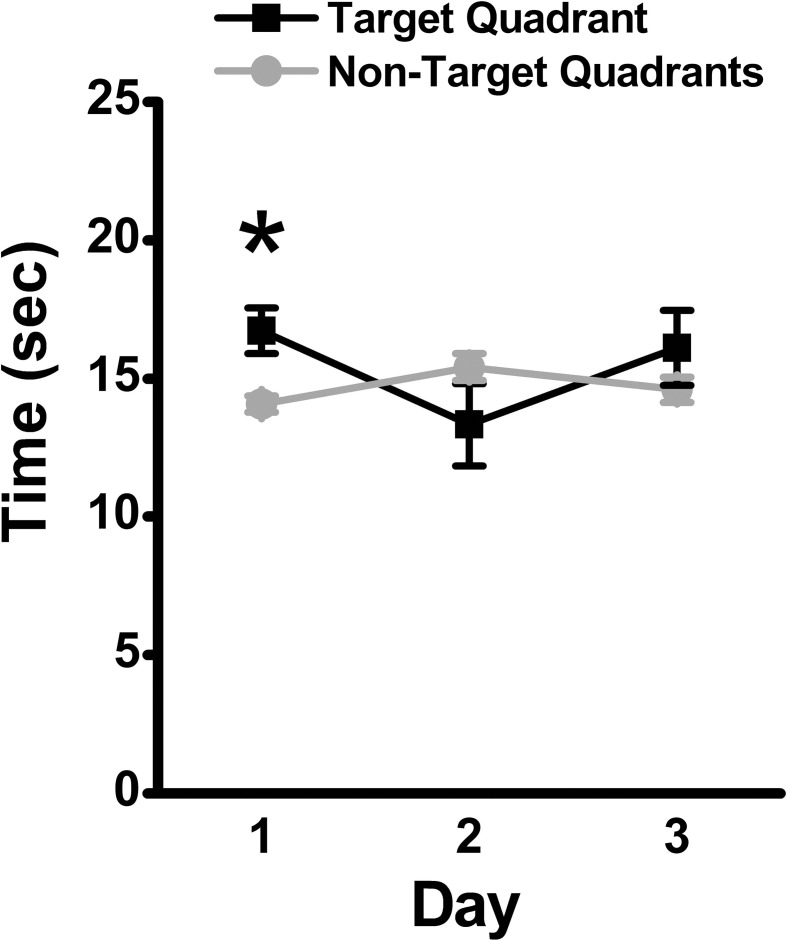
**Memory retention in the MWM assessed by time in target quadrant**. During the 3-day retention phase, the platform was removed, and the time spent in the target quadrant was compared to the average time spent in the non-target quadrants. Mean ± SEM; *n* = 10; ^*^p < 0.05.

Over the 3-day retention phase in which the platform was removed, used to assess memory for the previously learned platform location, the time spent in the target quadrant was compared to the average time spent in the non-target quadrants as an indicator of the animal's recall of the platform location. Mice spent significantly more time in the target quadrant as compared to the average time spent in the non-target quadrants on Day 1 (mean ± SEM: 16.73 ± 0.82 vs. 14.07 ± 0.31 s., respectively; *p* = 0.04), suggestive of recall of the platform location. The time spent in the target quadrant was not significantly differently from the average time spent in the non-target quadrants on Day 2 (mean ± SEM: 13.33 ± 1.48 vs. 15.4 ± 0.48 s, respectively; *p* > 0.05) or Day 3 (mean ± SEM: 16.1 ± 1.35 vs. 14.63 ± 0.46 s, respectively; *p* > 0.05) of the retention phase (Figure 3).

### Western blot data from the hippocampi of MWM-trained vs. untrained control mice

**Figure 4:**
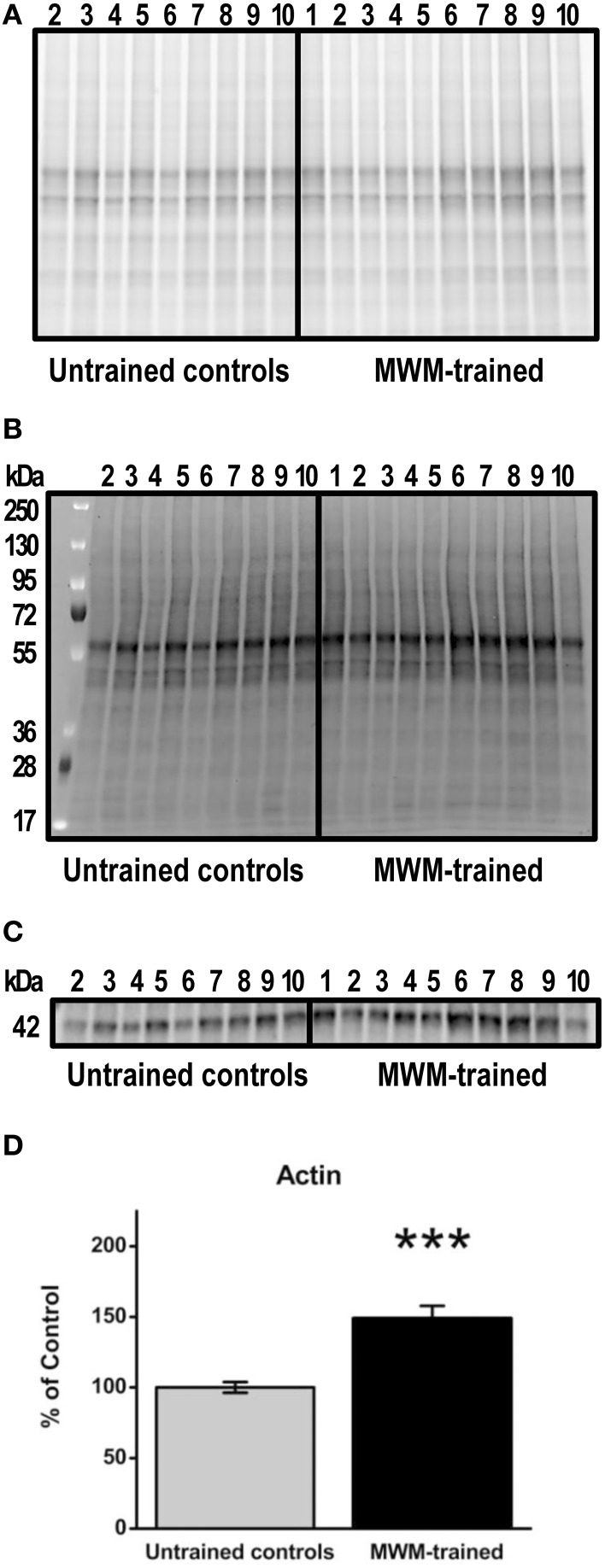
**Semi-quantification of actin protein levels in the hippocampus of MWM-trained vs. untrained control CD1 mice. (A)** Gel activation image of protein from hippocampal homogenates (15 μg) using UV illumination-based stain-free technology. **(B)** Image of nitrocellulose blot after protein transfer, indicating the total amount of protein in each lane that was used for normalization. **(C)** Representative Western blot detecting total actin (1:500) in hippocampal homogenates from MWM-trained and untrained control mice (*n* = 9–10). **(D)** Bar graph of densitometry values for actin, normalized to total protein and expressed as percentage change from control mean (100%) ± SEM, from hippocampal homogenates from untrained controls and MWM-trained mice. Error bars represent standard error; ^***^p < 0.001.

After MWM, brain tissue was extracted for Western blot experiments to detect transcription factor protein levels after training for comparison to levels in untrained controls. Prior to tissue freezing for Western blotting, hippocampi were weighed. No significant differences were found between untrained control (mean ± SEM: 0.054 ± 0.01) and MWM- trained mice (0.051 ± 0.01; *p* = 0.8; data not shown). Based on data normalized to total protein, hippocampal actin levels were significantly increased (*p* < 0.001; *n* = 9–10) in MWM-trained mice vs. untrained controls (Figure 4). This upregulation was confirmed in a repeated Western blot experiment (*p* = 0.04; *n* = 9–10; data not shown) and in an additional experiment in which group order was reversed (e.g., samples from MWM-trained mice loaded first; *p* = 0.01; *n* = 5) to rule out any systemic bias or artifact due to loading order (data not shown). Not surprisingly, no significant differences were detected in any of the transcription factors when normalized to actin levels (data not shown). Therefore, all subsequent immunoblot results were garnered with analyses based on densitometry values normalized to total protein (Figure 4B).

**Figure 5:**
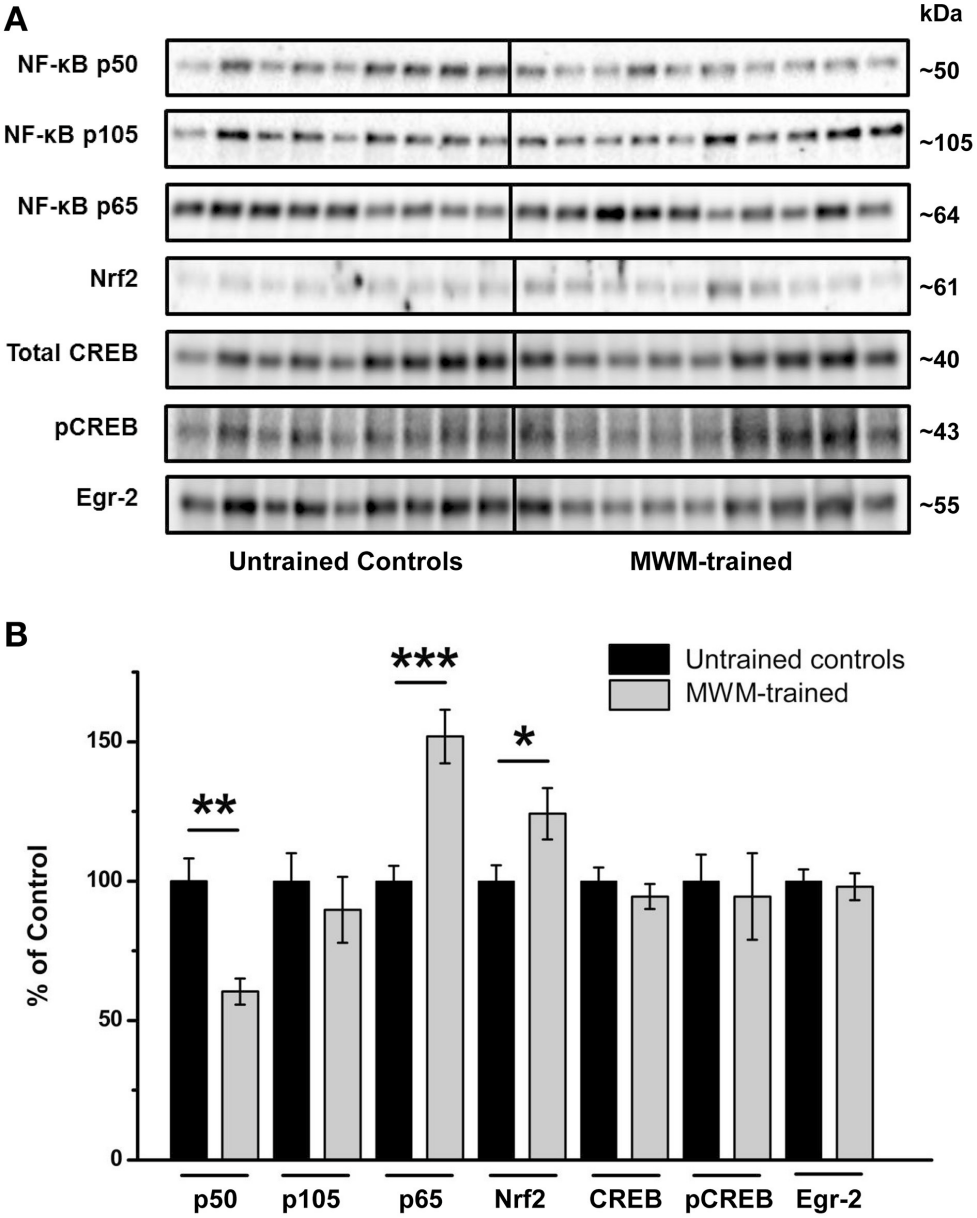
**Semi-quantification of transcription factor protein levels in the hippocampus of MWM-trained vs. untrained control CD1 mice**. **(A)** Representative Western blots detecting NF-κB subunits (p50, p105, and p65), Nrf2, CREB (total and activated pCREB), and Egr-2. All samples (*n* = 9–10/group) were immunoblotted simultaneously using 26-well Criterion™ TGX Stain-Free™ gels (Bio-Rad). **(B)** Bar graph of densitometry values of transcription factors of interest, normalized to total protein and expressed as percentage change from control mean (100%) ± SEM, from hippocampal homogenates from untrained controls and MWM-trained mice. ^*^p < 0.05; ^**^p < 0.01; ^***^p < 0.001.

Relative to untrained controls, levels of NF-κB subunit p50 were significantly lower in the hippocampi of mice after MWM training (*p* < 0.01) relative to untrained controls, with no significant differences in its precursor subunit, p105 (Figure 5). In contrast, levels of hippocampal NF-κB subunit p65 were significantly higher after MWM training compared to untrained controls (*p* < 0.001), as were Nrf2 protein levels (*p* < 0.05; Figure 5). No such differences, however, were found in hippocampal CREB, pCREB, or Egr-2 levels as a function of training (Figure 5).

### Associations between performance in the retention phase of the MWM and transcription factor levels

**Figure 6:**
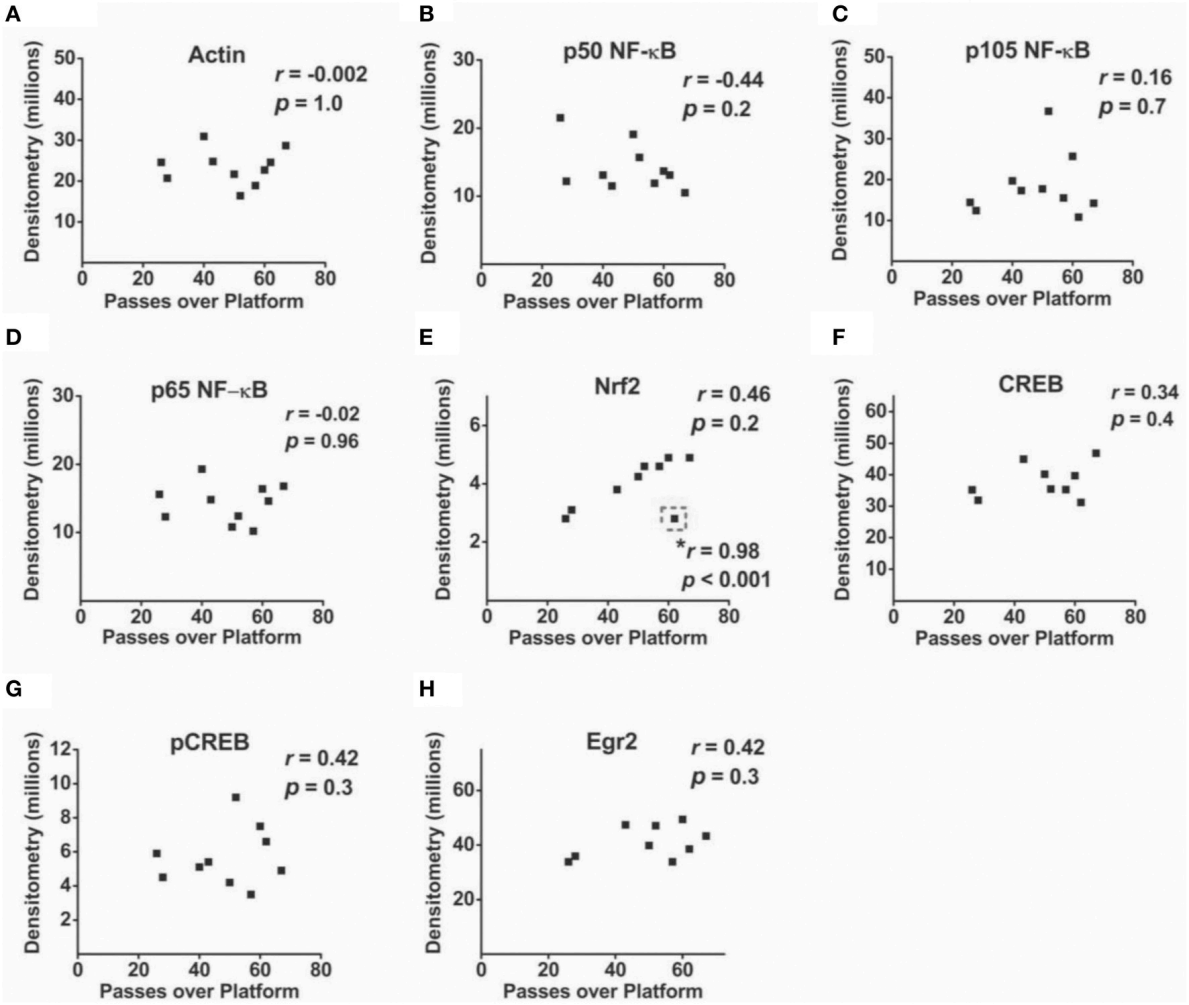
**Associations between hippocampal transcription factor protein levels and MWM-retention phase parameter passes over platform**. **(A–H)** Scatterplots and Pearson correlation coefficients between densitometry values (normalized to total protein) from Western blot experiments and the cumulative number of passes over the platform area during the MWM retention phase (*n* = 9–10). ^*^: highly significant positive correlation for *n* = 8∕9 tested animals after removal of one inconsistent data point (outlined in dashed line).

**Figure 7:**
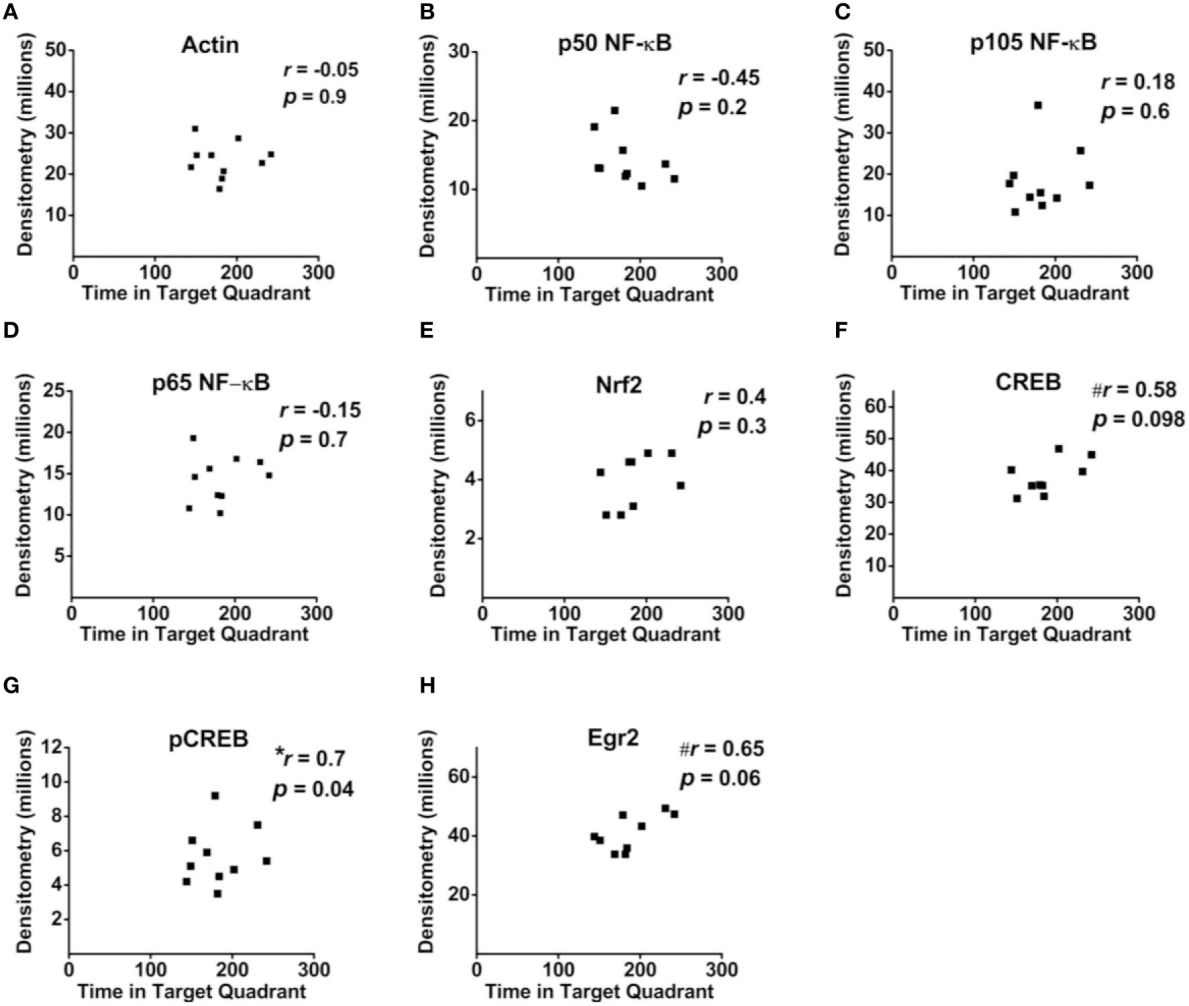
**Associations between hippocampal transcription factor protein levels and MWM-retention phase parameter time in target quadrant**. **(A–H)** Scatterplots and Pearson correlation coefficients between densitometry values (normalized to total protein) from Western blot experiments and the time spent in the target quadrant during the MWM retention phase. *n* = 9–10.

Correlational analyses between transcription factor densitometry values and cumulative passes over platform across the 3-day retention phase in trained mice did not reveal any significant associations (Figure 6). A correlation of 0.46 between Nrf2 and passes over platform, however, approached significance (*p* = 0.07). Examination of the scatterplot (Figure 6E) indicated the presence of a data point in which protein levels were low relative to passes over platform as compared to the trained group overall. Interestingly, a strongly positive significant correlation (*r* = 0.98; *p* < 0.01) was found between Nrf2 and passes over platform for the remaining eight animals with this inconsistent data point removed from the analysis. Therefore, in 88.8% of animals (*n* = 8∕9), the relationship between Nrf2 levels and passes over platform approached near perfect linearity. Correlational analyses using time spent in the target quadrant did not yield any significant relationships with actin (Figure 7A) or with any of the NF-κB subunits measured (Figures 7B–D). As well, Nrf2 was not significantly associated with this retention phase parameter (Figure 7E), in contrast to the significant relationship reported with passes over platform. Densitometry values for pCREB were significantly correlated with time in target quadrant in the positive direction (*r* = 0.7; *p* < 0.05; Figure 7G). The positive correlation (*r* = 0.58) between time in target quadrant and CREB levels approached significance (*p* < 0.1; Figure 7F), as was the case for time in target quadrant and Egr-2 levels (*r* = 0.65; *p* = 0.6; Figure 7H).

## Discussion

The present study simultaneously evaluated changes associated with MWM training in levels of three transcription factors previously implicated in memory. We hypothesized that training in a task associated with spatial learning and memory would alter levels of or be associated with regulators of activity-dependent plasticity, learning and memory, including NF-κB as well as CREB, and Egr-2, both of which are influenced by NF-κB dynamics. The effects of MWM training on Nrf2 were also analyzed to investigate training-induced transcriptional modifications of this master redox regulator. We found alterations in NF-κB and Nrf2, with no effect of training on CREB or Egr-2 levels. Interestingly, we found a decrease in NF-κB p50 levels after MWM training. NF-κB has multiple roles in the central nervous system, including inflammation regulation (Vallabhapurapu and Karin, 2009), development (Mincheva-Tasheva and Soler, 2013), synaptic plasticity, and learning and memory (Snow et al., 2014). Given its diverse functions in the brain, it is not surprising that neuronal NF-κB composition is complex, consisting of various dimers that can form from its multiple isoforms. Although we found downregulation of p50 NF-κB after training, this subunit is considered inhibitory, as p50 homodimers repress downstream gene expression (Ghosh and Karin, 2002). In line with such an inhibitory role is evidence demonstrating enhanced spatial learning in the MWM but not other tests of spatial learning (e.g., Barnes maze) in NF-κB-p50 KO mice (Lehmann et al., 2010). Increased levels of p65, however, may reflect enhanced activation, given that heterodimers consisting of p50 and p65, the most common dimer composition found in neurons, initiate gene expression in the nucleus (Mincheva-Tasheva and Soler, 2013). We cannot definitively state the implication of a reduction of the p50 subunit, given that its action on target genes (i.e., inhibition or activation) is a function of its associated subunit and resulting dimer composition. In addition to measures of NF-κB dimer subunits, future studies could measure levels of IκB to more accurately evaluate training-induced activation of NF-κB.

Relative to other organs, the brain has unusually high energy demands, exhibiting high oxygen consumption and robust production of reactive oxygen species (ROS). Studies have shown that formation and maintenance of LTP, a commonly studied cellular substrate for learning and memory, are ROS-dependent, including superoxide and H_2_O_2_ (Klann et al., 1998; Knapp and Klann, 2002). Furthermore, this regulation acts in a concentration-dependent manner (Kamsler and Segal, 2003). Therefore, ROS appear to be essential signaling components for memory formation; on the other hand, they can also impair the same neuronal networks necessary for memory function. Hence, it is logical to suggest the importance of transcriptional regulation of the redox state in memory formation. Nrf2 is a master transcriptional regulator of genes involved in antioxidant response and ROS production, and for some of these genes (SOD-1, NADPH oxidase), direct connections with hippocampus-dependent spatial memory function have already been documented (Gahtan et al., 1998; Kishida et al., 2006).

Memory improvement upon Nrf2 level modulation has been reported in aged APP/PS1 mice (Kanninen et al., 2009) and in rats with induced memory impairments (Dwivedi et al., 2013). To our knowledge, however, this is the first report to demonstrate alterations in hippocampal Nrf2 after training with a learning and memory paradigm in the typical mammalian brain. Further, we found a strong positive correlation between Nrf2 and performance in the memory retention phase of the MWM in the overwhelming majority of tested animals. Such data argue for the involvement of Nrf2 in hippocampal-mediated activity-dependent plasticity and memory.

Previous findings report opposing expression patterns between NF-κB p65 and Nrf2 (Ashabi et al., 2013; Permpoonputtana and Govitrapong, 2013; Tobón-Velasco et al., 2013; Djordjevic et al., 2015), in contrast to our findings of training-induced increases in both NF-κB p65 and Nrf2. These reports examined this relationship in cases of pathologies and putative treatments, where oxidative stress is an accompanying phenomenon. Therefore, our results suggest a dynamic regulation of NF-κB p65 and Nrf2 that appears condition-dependent, whereby components of these two transcription factors display similar expression patterns under physiological conditions but opposing patterns under pathological conditions.

In addition to elevations in Nrf2, this is the first study of which we are aware to report increased hippocampal actin levels after Morris water maze training in mice. Several lines of research confirm a role for actin, a fundamental component of the neuronal cytoskeleton, in synaptic plasticity, learning and memory. For instance, brain-specific KO of β-actin, the major actin isoform in the mammalian central nervous system, impaired performance in the MWM in mice, particularly in the memory retention phase (Cheever et al., 2012). Morphologically, sensitivity to β-actin depletion was region-specific, despite its ubiquitous presence in the brain. For example, the hippocampus and cerebellum were particularly vulnerable to the effects of a lack of β-actin, as gross morphology was aberrant. No such abnormalities were found in the cerebral cortex (Cheever et al., 2012). Thus, the hippocampus may be especially sensitive and responsive to actin levels in the context of both learning and memory formation and development.

Actin is intricately involved in the activity-dependent modulation of dendritic spines and synaptogenesis (Hotulainen and Hoogenraad, 2010). Actin rearrangement from monomeric (G-actin) to filamentous (F-actin) states via polymerization is required for consolidation of conditioned taste aversion memory (Bi et al., 2010) and conditioned place aversion memory (Hou et al., 2009). Pharmalogical inhibition of actin polymerization within the hippocampus impairs object placement memory formation in female rats in a dose-dependent fashion (Nelson et al., 2012). Further, interventions that induce actin rearrangement improve MWM performance in mice (Fu et al., 2014) without increasing total actin, confirming actin-induced activity-dependent synaptic remodeling in a manner independent of protein synthesis. Enlargement of the actin cytoskeleton and subsequent stabilization, however, may be considered a critical determinant of memory consolidation (Rudy, 2015). Others have reported activity-dependent upregulation of actin-binding proteins in the hippocampus, including increased levels of pCofilin after object placement training (Nelson et al., 2012). Our results of increased total actin protein levels suggest an additional mechanism of actin-induced changes, perhaps through increasing the available pool of hippocampal actin that would be required to induce spine formation and synaptogenesis. Indeed, Motanis and Maroun (2012) showed distinct physiological requirements of different phases of learning and memory. Specifically, they demonstrated that acquisition of contextual fear conditioning involves both actin rearrangement and protein synthesis, whereas reacquisition of fear conditioning after extinction was dependent upon actin rearrangement; protein synthesis was not required. Given data demonstrating behavioral consequences to changes in actin and our results of increased hippocampal actin in MWM-trained mice, the use of actin as a loading control in immunoblot studies investigating proteins putatively involved in activity-dependent plasticity, learning and memory should be undertaken with caution. Moreover, recent reports indicate less variable protein loading and increased linear range of detection with total protein as the loading control (Gilda and Gomes, 2013; Li and Shen, 2013), as in the present study, as compared to normalization with actin. Immunoblot studies investigating activity-dependent plasticity and/or the molecular basis of learning and memory should confirm whether or not differences in actin levels exist as a function of the experimental condition to determine its suitability as an internal control.

The role of CREB in learning and memory has been widely studied, with the general consensus being that CREB plays a pivotal role in learning and memory, including hippocampal-dependent spatial learning (Guzowski and McGaugh, 1997). Evidence suggests a role for CREB in LTM, but not short-term memory, in MWM tests (Florian et al., 2006). CREB signaling associated with learning and memory, however, has been shown to vary in magnitude as a function of brain structure and temporal dynamics, with CREB activation following a biphasic pattern post-training (Porte et al., 2008b). Further, the duration of activation of CREB may be more important in explaining hippocampal-mediated CREB activity associated with learning than the magnitude (Porte et al., 2008b).

Although, the literature supports a role for CREB in LTM, the view that CREB is *essential* for hippocampal synaptic plasticity and memory is not unanimous. Balschun et al. (2003) found only minimal impairment in the acquisition phase of the MWM, with no deficits in the memory retention phase in transgenic mouse in which the expression of CREB isoforms in the CA1 hippocampal subregion was disrupted. Further, they found no deficit in hippocampal LTP. Disruption of all CREB isoforms brain-wide failed to alter hippocampal LTP, LTD or contextual fear conditioning. Hippocampal-independent taste aversion conditioning, however, was severely compromised in mice lacking all CREB isoforms in the brain. In another study, blockade of all CREB isoforms in the CA1 hippocampal subfield did not alter late-phase LTP, the phase associated with memory consolidation/LTM (Pittenger et al., 2002). In this study, forskolin- and dopamine-associated LTP was diminished. Although the majority of studies across various species and experimental paradigms support a pivotal role for CREB in LTM, one possible explanation for reported inconsistencies is that, in some types of memory and/or under some conditions, memory formation may be CREB-independent (Alberini, 2009).

Interestingly, although we found decreases in NF-κB p50, we saw no changes in Egr-2, a gene target of NF-κB in neurons (Nafez et al., 2015), after MWM training. In contrast to our results with Egr-2, Egr-1 levels are up-regulated during spatial memory formation in the hippocampus (Pollak et al., 2005). The Egr family of proteins plays distinct roles in particular forms of memory (Poirier et al., 2008), with previous studies showing a paradoxical role for Egr-2 in learning and memory (Poirier et al., 2007). Egr-2-deficient mice show no impairment in spatial memory; rather, they exhibit improved performance in motor learning on rotarod tests and in object recognition memory tests (Poirier et al., 2007).

In the hippocampus, Egr-2 levels have a distinct spatial profile, with lower basal levels in the dentate gyrus relative to other subfields (Richardson et al., 1992). Other studies reveal subfield-specific differences in transcription factor levels after training. For example, although MWM-training upregulated pCREB and Egr-1, pCREB levels were highest in the CA3 region vs. CA1, whereas Egr-1 showed the reverse pattern (highest in the CA1 vs. CA3) (Zhou et al., 2013). Previous research has found no change in CREB levels after spatial learning, whereas pCREB increased (Porte et al., 2008a). Moreover, although training in the MWM induced CREB activation in the hippocampus overall, mice with the best recall of the platform location had the lowest levels of pCREB in the CA1 region (Porte et al., 2008b). The present study did not detect any differences in CREB as a function of MWM training. Performance in the memory retention phase was significantly correlated with pCREB levels but in the positive direction, unlike previous findings in the CA1 (Porte et al., 2008b). It must be noted, however, that the correlation with memory retention and pCREB levels, a finding that would not survive correction for multiple comparisons, was much weaker than that seen with Nrf2. The discrepancies in the literature regarding pCREB and memory may further be explained, in part, by the fact that we examined protein levels in homogenates from whole hippocampi, thus excluding an investigation of possible subfield-specific alterations induced by spatial learning in the MWM. Moreover, alterations in particular hippocampal transcription factors associated with memory in mice may be strain-dependent (Pollak et al., 2005). Further, not all strains of mice perform equally well in different mazes; some are better learners that others, and some perform better in specific mazes (Ammassari-Teule and De Marsanich, 1996; Crawley et al., 1997). These factors should be considered when evaluating reports of transcriptional regulation of learning and memory using mice as the model system.

Although the data presented herein demonstrate several protein alterations in the hippocampus after MWM training, indicating molecular changes associated with activity-dependent plasticity, the trained mice were exposed to significant stress, whereas the control were not. Hence, the effects of stress induced by the training paradigm on these changes cannot be ruled out. The significant correlations found for performance on the memory retention phase and Nrf2 protein levels, however, provide strong evidence implicating Nrf2 in hippocampal spatial memory formation, as do our findings of a significant association between pCREB levels and performance in the memory retention phase.

## Conclusions

In summary, this study revealed modifications in the levels of transcription factors associated with MWM-training in CD1 mice, including parallel elevations in NF-κB p65 and Nrf2, unlike the expression pattern seen in the literature thus far in pathological conditions. Egr-2 levels remained unchanged after training, as did CREB levels. A measure of memory retention was significantly correlated with Nrf2 in most animals. Actin was increased in MWM-trained animals relative to controls, thereby rendering actin an ineffective loading control in the present context and arguing for the use of total protein as an internal control, as presented here. These results support the view that training and performance in a spatial memory task are associated with transcriptional changes in the hippocampus, including those related to neuronal redox regulation.

## Author contributions

WS was involved in statistical analysis and interpretation of the data and writing the manuscript. PP was involved in carrying out the behavioral assays and dissections. JD was involved in experimental design of molecular data and manuscript writing. DM conducted western blot experiments and assisted with writing the manuscript. SA assisted with collecting behavioral data. EP and MB were involved in statistical analysis, interpretation, and writing the manuscript. MS assisted with editing the manuscript. BA was involved in conception and study design as well as manuscript writing.

### Conflict of interest statement

The authors declare that the research was conducted in the absence of any commercial or financial relationships that could be construed as a potential conflict of interest.

## Abbreviations

ANOVA: analysis of variance
CREB: cAMP-response element binding protein
Egr: early growth response
IKK: IκB kinase
KO: knock out
LTM: long-term memory
LTP: long-term potentiation
MW: molecular weight
MWM: Morris water maze
NF-κB: Nuclear factor kappa b
Nrf2: nuclear factor (erythroid-derived 2)-like 2
PIPES, piperazine-N: N′-bis (2-ethanesulfonic acid).
